# Supplementary material for: Complex‐centric proteome profiling by SEC‐SWATH‐MS
Source: Mol Syst Biol. 2019 Jan 14;15(1):e8438. doi: 10.15252/msb.20188438 (PMC6346213; doi:10.15252/msb.20188438)
Supplement: Supplementary file 7 — Dataset EV6 [file MSB-15-e8438-s007.zip › feature_plots_bioplex/Q7L2H7.pdf]

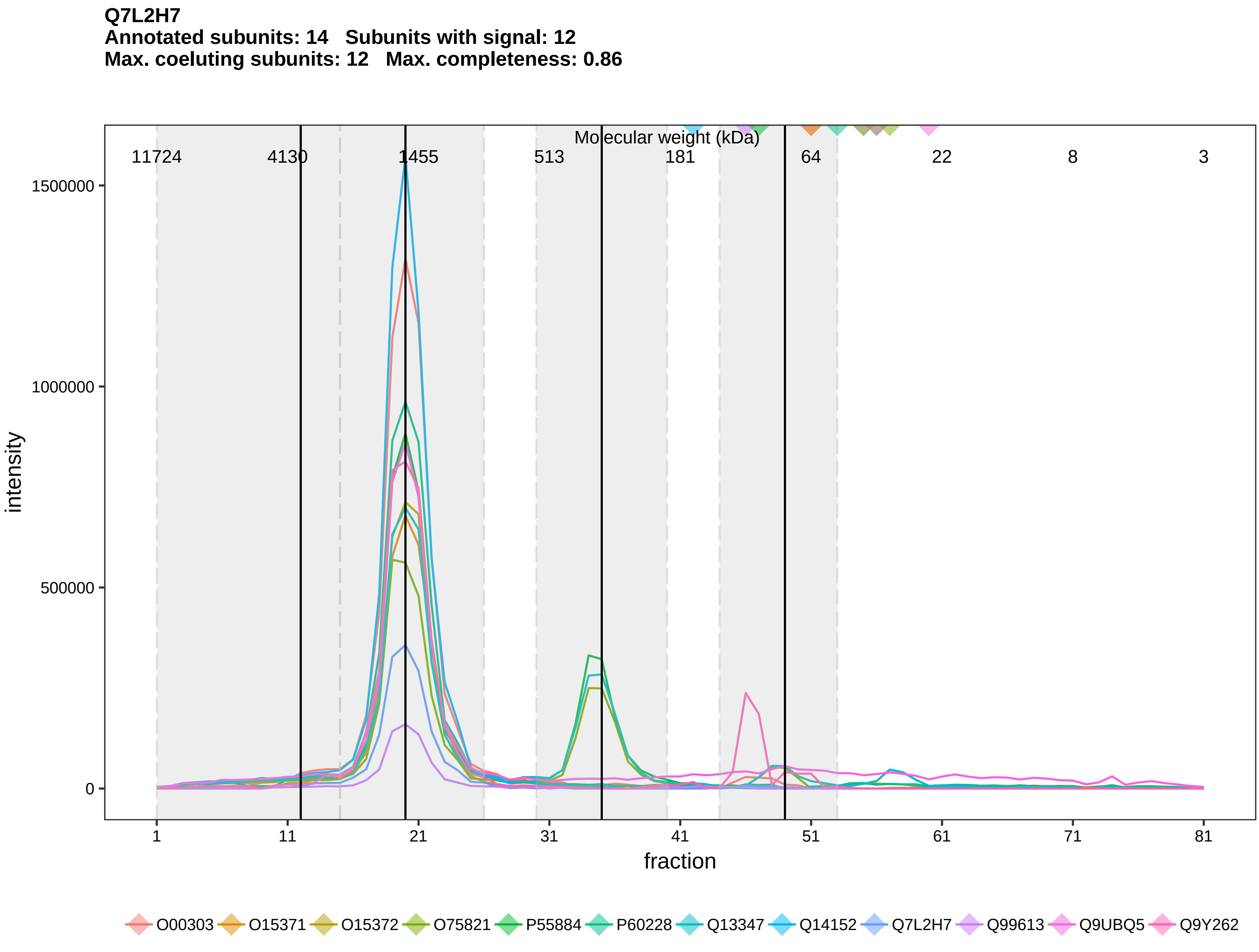
Q7L2H7
Annotated subunits: 14 Subunits with signal: 12
Max. coeluting subunits: 12 Max. completeness: 0.86
Molecular weight (kDa)
11724
4130
1455
513
181
64
22
8
3
1500000
1000000
500000
0
intensity
1
11
21
31
41
51
61
71
81
fraction
O00303
O15371
O15372
O75821
P55884
P60228
Q13347
Q14152
Q7L2H7
Q99613
Q9UBQ5
Q9Y262
